# Large-Scale Assessment of the Zebrafish Embryo as a Possible Predictive Model in Toxicity Testing

**DOI:** 10.1371/journal.pone.0021076

**Published:** 2011-06-28

**Authors:** Shaukat Ali, Harald G. J. van Mil, Michael K. Richardson

**Affiliations:** 1 Institute of Biology, Sylvius Laboratory, Leiden University, Leiden, The Netherlands; 2 Mathematical Institute, Leiden University, Leiden, The Netherlands; University of Birmingham, United Kingdom

## Abstract

**Background:**

In the drug discovery pipeline, safety pharmacology is a major issue. The zebrafish has been proposed as a model that can bridge the gap in this field between cell assays (which are cost-effective, but low in data content) and rodent assays (which are high in data content, but less cost-efficient). However, zebrafish assays are only likely to be useful if they can be shown to have high predictive power. We examined this issue by assaying 60 water-soluble compounds representing a range of chemical classes and toxicological mechanisms.

**Methodology/Principal Findings:**

Over 20,000 wild-type zebrafish embryos (including controls) were cultured individually in defined buffer in 96-well plates. Embryos were exposed for a 96 hour period starting at 24 hours post fertilization. A logarithmic concentration series was used for range-finding, followed by a narrower geometric series for LC_50_ determination. Zebrafish embryo LC_50_ (log mmol/L), and published data on rodent LD_50_ (log mmol/kg), were found to be strongly correlated (using Kendall's rank correlation tau and Pearson's product-moment correlation). The slope of the regression line for the full set of compounds was 0.73403. However, we found that the slope was strongly influenced by compound class. Thus, while most compounds had a similar toxicity level in both species, some compounds were markedly more toxic in zebrafish than in rodents, or vice versa.

**Conclusions:**

For the substances examined here, in aggregate, the zebrafish embryo model has good predictivity for toxicity in rodents. However, the correlation between zebrafish and rodent toxicity varies considerably between individual compounds and compound class. We discuss the strengths and limitations of the zebrafish model in light of these findings.

## Introduction

There is an unmet need for low-cost, high-throughput animal models in some fields of biomedical research such as drug screening and toxicity assessment [1], [2]. The zebrafish embryo is emerging as one such model [1]. It has been proposed as a bridge between simple assays based on cell culture, and biological validation in whole animals such as rodents [1]. The zebrafish cannot replace rodent models but is complementary to them, being particularly useful for rapid, high-throughput, low-cost assays, as for example in the early (pre-regulatory) stages of the drug development pipeline [3].

Among the attractive features of the zebrafish embryo model are its small size, small volume of compound consumed and rapid development. The organogenesis of major organs is completed at 5 days post fertilization (dpf) [4]. Also, many fundamental cellular and molecular pathways involved in the response to chemicals or stress are conserved between the zebrafish and mammals [5]. Genomic sequencing has shown extensive homology between zebrafish and other vertebrate species (including humans), and some aspects of brain patterning, structure and function are also conserved [6]–[9]. We have shown for example that the glucocorticoid receptor of the zebrafish is functionally closer to that of the human than is its mouse cognate [10]. The availability of genomic tools in the zebrafish provides an advantage over other teleosts such as the fathead minnow (*Pimephales promelas*) used, for example, in environmental toxicity assessment in the United States [11]. Indeed, zebrafish embryos may be a suitable replacement for some of these adult fish toxicity tests [12].

The zebrafish is increasing being used in toxicological studies [reviewed by: 13], [14]. Example include the use of adult zebrafish for the testing of lead and uranium [15], malathion [16], colchicine [17], anilines [18], and metronidazole [19]; and the use of juveniles for testing agricultural biocides [20]. Zebrafish embryos are also being used in toxicity studies [reviewed by: 21]. Examples include the use of zebrafish embryos for testing nanoparticles [22], [23].

Although the body plans of zebrafish are in many aspects similar to those of mammals, there are important differences. The fish is ectothermic, and lacks cardiac septa, synovial joints, cancellous bone, limbs, lungs and other structures [24]–[26]. Therefore, some toxic effects seen in humans are difficult to model in the zebrafish. Furthermore, the zebrafish embryo remains inside the chorion at least up to 48 hpf [27]. In pre-hatching embryos, therefore, the chorion (a membrane perforated by channels of 0.5–0.7 µm in diameter), may provide a barrier to diffusion of compounds [28]–[31].

The evolutionary divergence of zebrafish and mammals is around 445 million years ago [32] and so it is by no means certain that we will necessarily share the same sensitivity to toxic substances. Therefore, there is a need for validation of the model using compounds that have a known effect in other species [33]. One study has reported, using 18 toxic compounds, that toxicity in zebrafish was well-correlated with values reported from rodent studies [34]. The zebrafish embryo system has also been compared, as a toxicology screen, with the aquatic crustacean *Daphnia magna* [35]. Such studies are an important step towards the kind of comparative toxicity database represented by the well-known ‘Registry of Cytotoxicity’ which examines the predictive power of cell assays [36].

Our aim here is to determine the toxicity of 60 compounds from diverse pharmacological and chemical classes, and examine the strength of correlation between zebrafish embryo LC_50_ and data from the literature on rodent LD_50_. Compounds are added to the water in which the embryos develop, and so we focus here on water soluble compounds to avoid any confounding effects of carrier solvents.

## Materials and Methods

### Ethics statement

All animal experimental procedures were conducted in accordance with local and international regulations. The local regulation is the *Wet op de dierproeven* (Article 9) of Dutch Law (National) and the same law administered by the Bureau of Animal Experiment Licensing, Leiden University (Local). This local regulation serves as the implementation of *Guidelines on the protection of experimental animals* by the Council of Europe, Directive 86/609/EEC, which allows zebrafish embryos to be used up to the moment of free-living (approximately 5–7 days after fertilisation). Because embryos used here were no more than 5 days old, no licence is required by Council of Europe (1986), Directive 86/609/EEC or the Leiden University ethics committee.

### Animals

Male and female adult zebrafish (*Danio rerio*) of AB wild type were purchased from Selecta Aquarium Speciaalzaak (Leiden, the Netherlands) who obtain stock from Europet Bernina International BV (Gemert-Bakel, the Netherlands). Fish were kept at a maximum density of 100 individuals in glass recirculation aquaria (L 80 cm; H 50 cm, W 46 cm) on a 14 h light: 10 h dark cycle (lights on at 08.00). Water and air were temperature controlled (25±0.5°C and 23°C, respectively). The fish were fed twice daily with ‘Sprirulina’ brand flake food (O.S.L. Marine Lab., Inc., Burlingame, USA) and twice a week with frozen food (Dutch Select Food, Aquadistri BV, the Netherlands).

### Defined embryo buffer

To produce a defined and standardized vehicle for these experiments, we used 10% Hank's balanced salt solution (made from cell-culture tested, powdered Hank's salts, without sodium bicarbonate, Cat. No H6136-10X1L, Sigma-Aldrich, St Louis, MO) at a concentration 0.98 g/L in Milli-Q water (resistivity = 18.2 MΩ·cm), with the addition of sodium bicarbonate at 0.035 g/L (Cell culture tested, Sigma Cat S5761), and adjusted to pH 7.46. A similar medium has been used previously [37]–[39].

### Egg water

Egg water was made from 0.21 g ‘Instant Ocean®’ salt in 1 L of Milli-Q water with resistivity of 18.2 MΩ·cm.

### Embryo care

Eggs were obtained by random pairwise mating of zebrafish. Three adult males and four females were placed together in small breeding tanks (Ehret GmbH, Emmendingen, Germany) the evening before eggs were required. The breeding tanks (L 26 cm; H 12.5 cm, W 20 cm) had mesh egg traps to prevent the eggs from being eaten. The eggs were harvested the following morning and transferred into 92 mm plastic Petri dishes (50 eggs per dish) containing 40 ml fresh embryo buffer. Eggs were washed four times to remove debris. Further, unfertilized, unhealthy and dead embryos were identified under a dissecting microscope and removed by selective aspiration with a pipette. At 3.5 hpf, embryos were again screened and any further dead and unhealthy embryos were removed. Throughout all procedures, the embryos and the solutions were kept at 28±0.5°C, either in the incubator or a climatised room. All incubations of embryos were carried out under a light cycle of 14 h light: 10 h dark (lights on at 08.00). All pipetting was done manually, with an 8-channel pipetter.

### Viability of early embryos

There are reports of an early “mortality wave” in zebrafish embryos cultured under certain conditions [for examples], [ see: 40,41]. In order to assess this mortality wave in our facilities, and to avoid taking embryos during such a die-off, we raised cleaned embryos in 92 mm Petri dish (60 eggs per dish) containing 40 ml Hank's buffer alone, or egg water alone. We scored the fertilisation rate and mortality of embryos at 4, 8, and 24 hpf (see below) in these two media.

### Evaporation of buffer from 96-well plate

Evaporation rate of buffer from the 96-well plates (Costar 3599, Corning Inc., NY) was determined as follows. In each well of the plate, 250 µL of freshly prepared buffer was dispensed at 0 h. As for all 96-well plate experiments reported in this study, the lids were in place but were not sealed with a sealing mat or film (because preliminary studies indicated that all embryos die within sealed plates). The plates were kept at 28±0.5°C without refreshing the buffer (static non-replacement regime) and weighed at daily intervals on a digital balance. Results were calculated as mean from four different plates. Buffer volume from some individual wells in different regions of the plate were also weighed at 4 days to determine the impact of well location on the evaporation rate.

### Test compounds

We used water-soluble compounds representing a range of different chemical classes and biochemical activities (Table S1). The required dilution was always freshly prepared in buffer just prior to assay on zebrafish embryos.

### Mortality scoring

Mortality rate (Table 1) was recorded at 48, 72, 96 and 120 hpf in both logarithmic series and geometric series using a dissecting stereomicroscope. Embryos were scored as dead if they were no longer moving, the heart was not beating and the tissues had changed from a transparent to an opaque appearance.

**Table 1:** Concentration-dependent mortality at 5 dpf after 96 h exposure.

		Cumulative % mortality after 96 h exposure										
	Compounds	logarithmic series (mg/L)†					geometric series* ± SEM					
		0	1	10	100	1000	C0	C1	C2	C3	C4	C5
1	Aconitine	0	0	0	63	100¥	0±0	67±2	98±1	100±0	100±0	100±0
2	Atropine	0	0	0	0	100	0±0	0±0	0±0	17±0	73±1	100±0
3	Berberine chloride	0	0	0	25	100	0±0	0±0	17±2	54±4	100±0	100±0
4	Colchicine	0	0	0	100	100	0±0	0±0	4±1	42±2	98±1	100±0
5	Coniine	0	0	0	100	100	0±0	0±0	0±0	2±1	100±0	100±0
6	α-Lobeline hydrochloride	0	0	0	100	100	0±0	0±0	6±2	83±2	100±0	100±0
7	Morphine hydrochloride	0	0	0	0	0	0±0	0±0	0±0	0±0	25±0	94±0
8	Nicotine	0	0	0	100	100	0±0	4±1	8±1	54±4	100±0	100±0
9	Quinine sulfate	0	0	0	88	94	0±0	0±0	0±0	0±0	0±0	42±1
10	(−)-Scopolamine hydrobromide trihydrate	0	0	0	6	6	0±0	0±0	2±1	4±1	19±1	77±2
11	Strychnine hydrochloride	0	0	0	100	100	0±0	29±2	40±1	67±1	100±0	100±0
12	Theobromine	0	0	0	50	100¥	0±0	13±2	15±2	38±0	58±4	100±0
13	(+)-Tubocurarine chloride hydrate	0	0	0	0	100	0±0	0±0	6±0	35±1	100±0	100±0
14	Yohimbine hydrochloride	0	0	0	75	100	0±0	13±2	13±2	23±1	29±1	81±0
15	Amygdalin	0	0	25	94	100	0±0	0±0	2±1	8±1	17±3	40±2
16	Arbutin	0	0	0	100	100	0±0	0±0	9±1	48±7	50±6	67±5
17	Convallatoxin	0	0	0	78	100¥	0±0	69±5	78±5	96±1	100±0	100±0
18	Coumarin	0	0	0	0	100	0±0	17±2	23±3	40±1	98±1	100±0
19	Digitoxin	0	25	100	100¥	100¥	0±0	27±1	94±1	100±0	100±0	100±0
20	Gentamycin sulfate	0	0	0	6	100	0±0	29±1	34±1	67±1	92±0	92±0
21	Glycyrrhizin	0	0	6	100	100	0±0	0±0	12±1	35±2	69±6	94±1
22	Hesperidin	0	0	0	69	100¥	0±0	0±0	8±2	10±3	63±1	81±2
23	Kanamycin monosulfate	0	6	13	38	38	0±0	2±1	2±1	15±1	46±1	79±5
24	Naringin	0	0	0	63	94	0±0	0±0	2±1	6±2	10±2	77±6
25	Neohesperidin	0	0	0	100	100¥	0±0	0±0	0±0	0±0	0±0	34±1
26	Ouabain octahydrate	0	0	0	19	100	0±0	2±1	6±1	65±3	96±1	96±1
27	Phloridzin dihydrate	0	0	0	0	100	0±0	0±0	2±1	6±1	12±1	65±3
28	Rutin hydrate	0	0	0	0	0	0±0	0±0	8±2	8±2	10±1	73±6
29	Streptomycin sulfate	0	0	0	6	31	0±0	0±0	0±0	0±0	13±0	73±1
30	Cadmium(II) chloride	0	38	38	100	100	0±0	19±0	25±1	60±3	84±1	100±0
31	Copper(II) nitrate trihydrate	0	0	13	100	100	0±0	0±0	2±1	13±0	38±0	100±0
32	Lead acetate trihydrate	0	0	0	94	100	0±0	25±6	33±8	35±8	94±1	94±1
33	Lithium chloride	0	0	0	0	0	0±0	0±0	15±1	60±5	100±0	100±0
34	Chloramphenicol	0	0	0	0	94	0±0	0±0	0±0	12±1	94±1	100±0
35	Ethanol	0	0	0	0	0	0±0	0±0	0±0	0±0	0±0	21±1
36	Glycerol	0	0	0	0	0	0±0	0±0	0±0	0±0	0±0	98±1
37	Tween 80	0	0	0	0	100	0±0	0±0	2±1	71±4	100±0	100±0
38	Acetic acid	0	0	0	38	100	0±0	0±0	4±1	67±1	100±0	100±0
39	Salicylic acid	0	0	6	100	100	0±0	0±0	0±0	10±2	100±0	100±0
40	Sodium oxalate	0	0	0	0	94	0±0	0±0	33±2	52±1	77±3	98±1
41	Trichloroacetic acid	0	0	6	56	100	0±0	0±0	33±8	60±20	100±0	100±0
42	Ampicillin sodium	0	0	0	0	38	0±0	0±0	0±0	19±0	19±0	35±1
43	Cyclophosphamide monohydrate	0	0	0	0	0	0±0	0±0	73±2	96±1	100±0	100±0
44	Paracetamol	0	0	0	0	100	0±0	0±0	0±0	8±1	90±10	100±0
45	Phenacetin	0	0	0	0	94	0±0	0±0	0±0	2±1	83±2	100±0
46	Benserazide hydrochloride	0	0	0	0	6	0±0	0±0	2±1	8±2	31±2	86±3
47	Chlorpromazine hydrochloride	0	0	94	100	100	0±0	0±0	0±0	4±1	67±1	100±0
48	Isoniazid	0	0	0	6	38	0±0	0±0	2±1	2±1	67±4	94±2
49	Phenelzine sulfate	0	0	19	100	100	0±0	4±1	23±1	100±0	100±0	100±0
50	Ethambutol dihydrochloride	0	0	0	0	0	0±0	0±0	0±0	17±3	73±4	100±0
51	Verapamil hydrochloride	0	0	0	100	100	0±0	0±0	2±1	10±2	42±7	100±0
52	Phenol	0	0	0	100	100	0±0	0±0	0±0	0±0	38±2	100±0
53	Sodium azide	0	100	100	100	100	0±0	0±0	10±2	90±3	100±0	100±0
54	Dimethyl sulfoxide	0	0	0	0	0	0±0	0±0	0±0	0±0	4±1	100±0
55	Formaldehyde	0	0	50	100	100	0±0	0±0	0±0	15±1	71±1	100±0
56	Phenformin hydrochloride	0	0	0	13	100	0±0	2±1	6±2	17±2	92±2	100±0
57	Ropinirole hydrochloride	0	0	0	0	100	0±0	8±1	8±1	21±1	96±1	100±0
58	Amitriptyline hydrochloride	0	0	63	100	100	0±0	4±1	6±2	40±2	100±0	100±0
59	Sodium dodecyl sulfate	0	0	94	100	100	0±0	8±±2	33±8	58±5	92±1	100±0
60	Barbital sodium	0	0	0	0	6	0±0	0±0	0±0	13±3	50±0	90±3

Key:

(†) This was a one-time range-finding experiment and so there is no SEM;

(*) a different geometric scale was used for different compounds because of the variations in toxicity found with the logarithmic range-finding. The values given are the mean percentage mortality from three replicates; the geometric series concentrations C0, C1, etc. are given for each compound in Table S2. For each concentration for each compound, N = 48 (3 replications x16) embryos;

(¥) percentage mortality was found but at these high concentrations, compounds were precipitated out of solution.

### Range-finding

To determine a suitable range of concentrations for testing, we performed range-finding using a logarithmic series (0.0, 1.0, 10.0, 100.0 and 1000 mg/L) as recommended in standard protocols [11]. Zebrafish embryos of 24 hpf from Petri dish were gently transferred using a sterile plastic pipette into 96-well microtitre plates. A single embryo was plated per well, so that dead embryos would not affect others, and also to allow individual embryos to be tracked for the whole duration of the experiment. A static non-replacement regime was used. Thus there was no replacement or refreshment of buffer after the addition of compound. Each well contained 250 µL of either freshly prepared test compound; or vehicle (buffer) only as controls. We used 16 embryos for each concentration and 16 embryos as controls for each drug.

### Geometric series and LC_50_ determination

After the range finding experiments, a series of concentrations lying in the range between 0% and 100% mortality were selected. The actual concentrations used are shown in Table S2. The concentrations were in a geometric series in which each was 50% greater than the next lowest value [11]. Each geometric series of concentrations for each compound was repeated three times (in total 48 embryos per concentration and 48 embryos for vehicle for each drug). LC_50_ (expressed in mg/L of buffer) was determined based on cumulative mortality obtained from three independent experiments at 120 hpf using Regression Probit analysis with SPSS Statistics for windows version 17.0 (SPSS Inc., Chicago, USA). Thus the embryos are exposed to the drug for 96 h. The LC_50_ in mg/L was converted into LC_50_ mmol/L to make relative toxicity easier to examine.

### Rodent data

The sources of LD_50_ data from rodents (rats and mice) are shown in Table 2.

**Table 2:** Zebrafish embryo LC_50_ values found in this study, and the corresponding rodent LD_50_ values based on the literature.

	Compounds	Zebrafish Embryo LC_50_ (mg/L ±SEM)	Zebrafish Embryo LC_50_ (mmol/L ±SEM)	Rodent LD_50_ (mg/kg)	Rodent LD_50_ (mmol/kg)
1	Aconitine	34.3±1.5	0.05±0.00	1(*)	0.002
2	Atropine	607.8±7.7	2.10±0.03	500(*)	1.73
3	Berberine chloride	129.2±3.6	0.35±0.01	60(*)	0.16
4	Colchicine	41.5±0.7	0.10±0.00	5.9(*)	0.02
5	Coniine	55.1±0.2	0.43±0.00	80(*)	0.63
6	α-Lobeline hydrochloride	30.9±0.9	0.08±0.00	39.9(*)	0.11
7	Morphine hydrochloride	9915.1±0.8	23.39±0.00	745(*)	1.76
8	Nicotine	35.1±0.5	0.22±0.00	50(#)	0.31
9	Quinine sulfate	562.4±9.5	1.44±0.02	800(*)	2.04
10	(−)-Scopolamine hydrobromide trihydrate	11465.1±166.1	26.16±0.38	1413(*)	3.22
11	Strychnine hydrochloride	20.8±0.58	0.06±0.00	2.73(*)	0.01
12	Theobromine	150.4±1.83	0.83±0.01	530(*)	2.94
13	(+)-Tubocurarine chloride hydrate	414.2±3.6	0.61±0.01	33(*)	0.05
14	Yohimbine hydrochloride	93.0±1.5	0.24±0.00	55(*)	0.14
15	Amygdalin	268.5±19.6	0.59±0.04	250(*)	0.55
16	Arbutin	120.9±13.0	0.44±0.05	500(*)	1.84
17	Convallatoxin	36.6±3.5	0.07±0.01	15.2(*)	0.03
18	Coumarin	241.2±8.9	1.65±0.06	293(*)	2.01
19	Digitoxin	0.5±0.06	0.001±0.00	4.1(*)	0.01
20	Gentamycin sulfate	253.3±6.5	0.44±0.01	384(*)	0.67
21	Glycyrrhizin	55.8±3.0	0.07±0.00	589(*)	0.70
22	Hesperidin	77.6±3.2	0.13±0.01	1000(*)	1.64
23	Kanamycin monosulfate	1787.5±16.8	3.07±0.03	1700(*)	2.92
24	Naringin	850.1±78.5	1.46±0.14	2000(*)	3.45
25	Neohesperidin	199.5±1.2	0.33±0.00	1000(*)	1.64
26	Ouabain octahydrate	184.1±4.8	0.25±0.01	3.75(*)	0.01
27	Phloridzin dihydrate	793.2±5.1	1.68±0.01	500(*)	1.06
28	Rutin hydrate	8722.9±164.24	14.29±0.27	2000(*)	3.28
29	Streptomycin sulfate	3164.0±35.4	2.17±0.02	600(*)	0.41
30	Cadmium(II) chloride	27.9±0.1	0.06±.00	88(#)	0.18
31	Copper(II) nitrate trihydrate	58.7±1.2	0.24±0.00	940(*)	3.89
32	Lead acetate trihydrate	62.4±1.1	0.16±0.00	174(*)	0.46
33	Lithium chloride	3324.2±143.6	78.42±3.39	1165(*)	27.48
34	Chloramphenicol	525.0±7.4	1.62±0.02	400(*)	1.24
35	Ethanol	36212.0±501.8	786.02±10.89	14008.3(#)	304.07
36	Glycerol	23357.4±282.1	253.58±3.06	12619(#)	137.00
37	Tween 80	323.4±10.1	0.25±0.01	25021(#)	19.10
38	Acetic acid	186.3±1.0	3.10±0.02	3309.3(#)	55.11
39	Salicylic acid	46.7±1.2	0.34±0.01	184(*)	1.33
40	Sodium oxalate	372.2±2.9	2.78±0.02	155.4(#)	1.16
41	Trichloroacetic acid	66.4±4.7	0.41±0.03	270(*)	1.65
42	Ampicillin sodium	6068.5±114.9	16.34±0.31	5314(*)	14.31
43	Cyclophosphamide monohydrate	1777.4±26.1	6.37±0.09	1930.9(#)	6.92
44	Paracetamol	535.8±17.1	3.54±0.11	367(*)	2.43
45	Phenacetin	309.9±8.4	1.73±0.05	634(*)	3.54
46	Benserazide hydrochloride	4747.9±28.7	16.17±0.10	5000(*)	17.02
47	Chlorpromazine hydrochloride	7.0±0.04	0.02±0.00	20(*)	0.06
48	Isoniazid	1297.5±38.0	9.46±0.28	1250(*)	9.12
49	Phenelzine sulfate	11.5±0.13	0.05±0.00	125(*)	0.53
50	Ethambutol dihydrochloride	6325.9±197.2	22.82±0.71	6800(*)	24.53
51	Verapamil hydrochloride	81.1±4.8	0.17±0.01	108(#)	0.22
52	Phenol	86.4±0.8	0.92±0.01	112(*)	1.19
53	Sodium azide	1.4±0.04	0.02±0.00	19(*)	0.29
54	Dimethyl sulfoxide	20964.6±158.1	268.33±2.02	19691.3(#)	252.03
55	Formaldehyde	12.7±0.1	0.42±0.00	42(*)	1.40
56	Phenformin hydrochloride	508.3±17.6	2.10±0.07	407(*)	1.69
57	Ropinirole hydrochloride	437.3±10.2	1.47±0.03	396(*)	1.33
58	Amitriptyline hydrochloride	8.0±0.1	0.03±0.00	21(*)	0.07
59	Sodium dodecyl sulfate	3.6±0.3	0.01±0.00	118(*)	0.41
60	Barbital sodium	3902.5±30.5	18.93±0.15	3101(#)	15.04

Key:

(*) from Chemical Identification/Dictionary database at http://toxnet.nlm.nih.gov/cgibin/sis/search/;

(#) from [36].

### Statistical analysis

Statistical analyses were performed using GraphPad Prism for Windows (version 5.03) or R (v. 2.12). One way ANOVA and Newman-Keuls Multiple Comparison test was employed for survival rate. Correlation and ANCOVA models were used to investigate the relationship between LC_50_ in zebrafish embryos and published LD_50_ values in rodents.

## Results and Discussion

We have examined the toxicity, in zebrafish embryos, of a 96 h exposure (during the period 24 hpf to 5 dpf) to 60 compounds of differing biochemical classes. Our logarithmic and geometric concentration series both showed concentration-dependent mortality. LC_50_ values were determined, and compared with rodent LD_50_ values from the literature.

### Infertility and spontaneous early mortality of eggs/embryos

**Figure 1:**
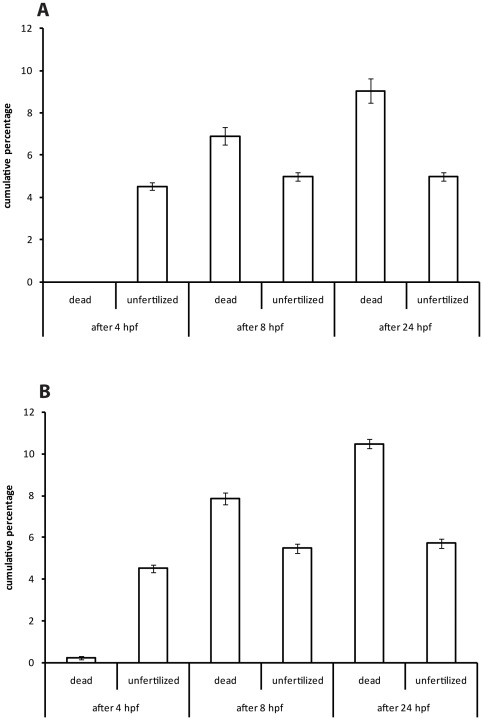
Cumulative mortality and infertility of zebrafish in buffer or egg water. Embryos were kept in 92 mm Petri dishes with 40 ml of either buffer or egg water, 60 eggs per dish. Each error bar represents ±SEM of N = 420 embryos each for buffer and egg water. **A**, cumulative infertility and early mortality in buffer. **B**, the same, in egg water. There is no significant difference between the two media in terms of survival and fertilization percentage.

We found that, in controls (buffer only), 5% of eggs were unfertilised, and a further 9% represented embryos that died spontaneously in the first 24 hpf. This is similar to the spontaneous mortality of 5–25% reported elsewhere for early zebrafish development [40]. We find no significant difference between these values when Hank's buffer was used as the medium, and when egg water was used (Figure 1A, B). In order to avoid this natural early mortality we began our assays at 24 hpf. This also makes our study consistent with a previous one, in which the zebrafish was exposed to different compounds at 24 hpf to find the correlation between zebrafish and rodent toxicities [34].

It could be argued that, by beginning exposure at 24 h, we are missing out on early developmental toxicity effects, such as the action of compounds on gastrula stages. However, this is likely to be a trade-off because other compounds mainly cause embryo death at these early stages. For example, a recent study [42] showed that exposure of zebrafish embryos at early stages (*dome* to *26-somite*) to ethanol resulted in high mortality, while exposure at later stages (*prim-6* and *prim-16*) led to a high incidence of abnormal embryos. Other examples of compounds which are more toxic to larval stages than to embryonic and adult stages of freshwater fish species are copper and cadmium [43]–[45]. Finally, it is known that presence of chorion at early stages acts as a possible barrier to diffusion of compounds [29], [30], [42].

### Rate of evaporation from 96-well plates at 28.0°C

**Figure 2:**
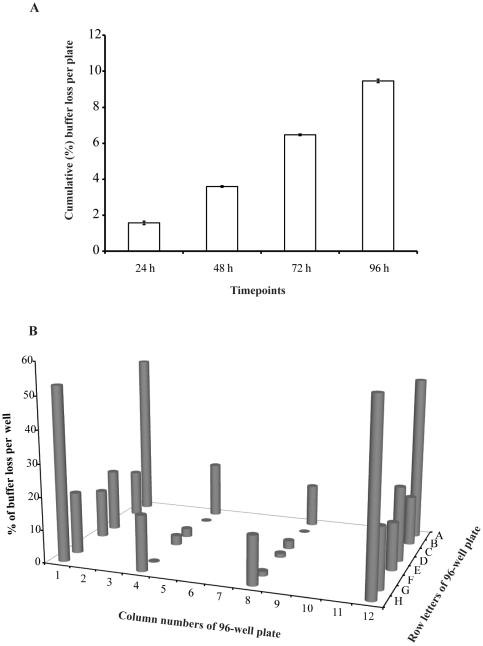
Rate of evaporation from 96-well plates at 28.0°C. Buffer was dispensed in four different 96-well plates. **A**, cumulative average percentage buffer loss per plate. All wells were initially filled with 250 µL buffer. **B**, percentage buffer loss after 96 h, per well, as a function of well position. The letters A–H and the numbers 1–12 correspond to the standard coordinates embossed into 96-well plates. All wells were initially filled with 250 µL buffer. Only the wells with grey columns were measured.

In our study, we did not replace the buffer. Therefore, we decided to check how much water would be lost during this period by evaporation from the 96-well plate (with its lid in place). We found that, by 96 h of incubation at 28.0°C, 9.46% of the buffer had evaporated (Figure 2A). Further investigation showed that the rate of evaporation was higher in the external rows and columns, and highest of all in the four corner wells (Figure 2B). In view of this evaporation pattern, we filled all the 96-wells with buffer, but did not plate embryos into wells A1-H1 and A12-H12. A way of mitigating the effects of this rate of evaporation would be to use dynamic replacement of buffer, as in a microfluidic chip [39], or static replacement (e.g. daily refreshing). Nonetheless, static non-replacement, as used here, is a popular technique for zebrafish embryo culture, and was used in a recent toxicity study [46].

### Concentration response and LC_50_ of compounds

For all compounds, mortality at 5 dpf was concentration-dependent (Table 1). This was true for both logarithmic and geometric series. By contrast, controls (vehicle only) showed 0% mortality. The LC_50_ values are shown in Table 2.

### Correlation between zebrafish embryo log LC_50_ and rodent log LD_50_

**Figure 3:**
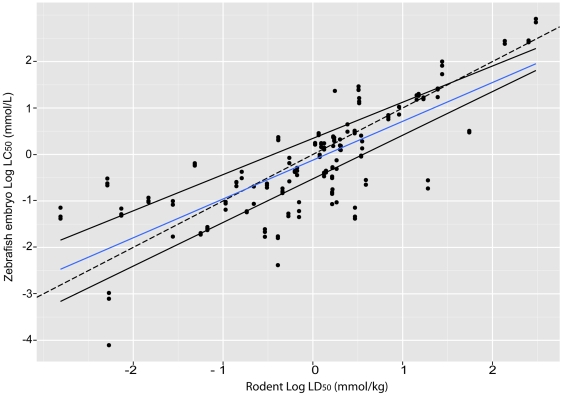
Correlation between zebrafish embryo Log LC_50_ and rodent Log LD_50_ for the 60 compounds tested in this study. Zebrafish embryo LC_50_ was determined based on cumulative mortality after 96 h exposure of compounds from three independent experiments and rodent LD_50_ was taken from the literature. Key: blue, regression line; solid black lines, 0.25 and 0.75 quartiles; dashed line, perfect correlation line. The slope of the regression line (blue) is 0.73403.

To examine the ability of zebrafish assays to predict toxicity in rodents, we analysed a correlation between our zebrafish embryo log LC_50_ values, and rodent log LD_50_ from the literature. The comparison is shown graphically in Figure 3. A correlation test produced Spearman's rank correlation of 0.7688 (p<0.001) and a Pearson's product-moment correlation 0.7832 (df = 178, p<0.001) between zebrafish embryo LD_50_ and rodent log LD_50_ for the whole set of compounds. These values of correlation indicate that zebrafish LC_50_ and rodent LD_50_ values co-vary. This is consistent with a previous report [34] that the toxicity of 18 compounds in zebrafish embryos was well-correlated with values reported from rodent studies. It is also in line with another study [46] suggesting that zebrafish embryos could be used as a predictive model for the developmental toxicity of compounds.

### Toxicity by compound class

**Figure 4:**
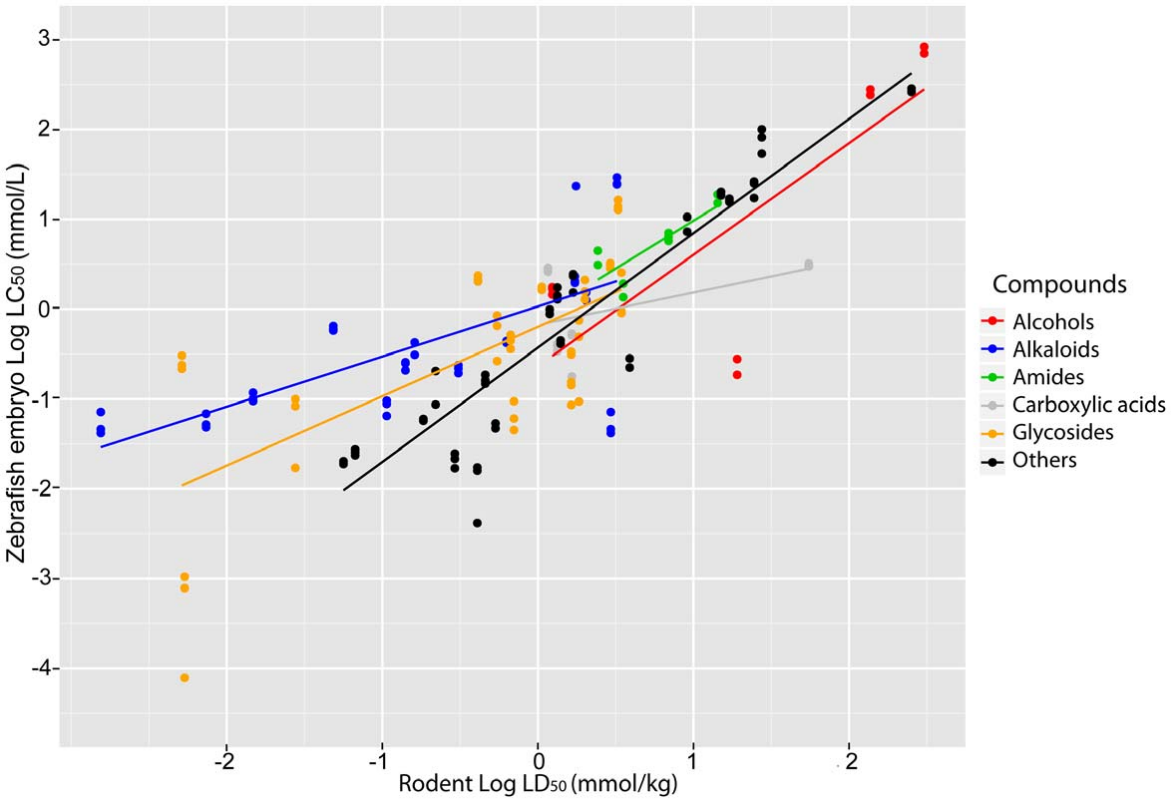
Linear regression model: rodent log LD_50_ and zebrafish embryo log LC_50_. The effect of the different compounds on the slope and intercept of the ANCOVA model. Although we must consider the effect of the unknown error in the rodent LD_50_ values, the different compound classes seem to cluster in different regions in the graph.

We next developed a statistical model that examines the similarity between zebrafish and rodent toxicity values when the compounds are clustered into chemically similar groups. To do this, we mapped zebrafish values to rodent values, taking account of specific variances in intercept and slope, due to those groupings. The groupings were alcohols, alkaloids, amides, carboxylic acids, glycosides and the remaining compounds (others). We designed an ANCOVA with the values [zebrafish embryo log LC_50_] as dependent variables, and [rodent log LD_50_] and [compound type] as independent variables. Table 3 shows the statistics of our ANCOVA model, while the dataset is displayed graphically in Figure 4. As can be seen, there is a significant effect of compound type on intercept and slope.

**Table 3:** Statistical analysis of regression per group of compound using the ANCOVA model described in the text.

Coefficients	Estimate	Std. error	t-value	p-value	Significance level
Intercept: Others	−0.43	0.08704	−4,930	1.96E-06	#
Intercept: Alcohols	−0.64	0.35187	−1,813	0.071546	
Intercept: Alkaloids	0.03	0.11947	0.24	0.810735	
Intercept: Amides	−0.08	0.49548	−0.168	0.866425	
Intercept: Carboxylic acids	−0.17	0.23275	−0.751	0.45351	
Intercept: Glycosides	−0.2	0.10027	−1,970	0.050426	
Slope: Others	1.27	0.08803	14,456	2.00E-16	*
Slope: Alcohols	1.24	0.21852	−0.139	0.889249	*
Slope: Alkaloids	0.56	0.13171	−5,427	1.97E-07	*
Slope: Amides	1.06	0.63408	−0.326	0.744924	
Slope: Carboxylic acids	0.36	0.27869	−3,279	0.001265	*
Slope: Glycosides	0.77	0.13576	−3,684	0.000309	*

The slope for amides (Table 3) does not differ significantly from 1.0, indicating a very similar toxicity in zebrafish and rodents. By contrast, ‘others’ and alcohols have a slope significantly greater than 1.0, indicating that they are generally less toxic in zebrafish than in rodents. The groups carboxylic acids, glycosides and alkaloids have a slope significantly less than 1.0 indicating that they are more toxic in zebrafish than in rodents (Table 3).

If we look at the relative toxicity ([zebrafish LC_50_ mmol/L] ÷ [rodent LD_50_ mmol/kg]) of individual compounds we see the following examples of compounds that have a similar toxicity in the two sepcies: coumarin (0.95), benserazide hydrochloride (1.06), phenformin hydrochloride (1.11) and theobromine (1.11). Examples of compounds less toxic in zebrafish than in rodents are aconitine (0.01), ouabain octohydrate (0.02), tubocurarine hydrochloride (0.07), morphine hydrochloride (0.08) and colchicine (0.13). At the other extreme are compounds more toxic in zebrafish than in rodents including: Tween80 (103.01), sodium dodecyl sulfate (98.33), lead acetate trihydrate (29.49) and copper (II) nitrate trihydrate (19.40).

Among the alcohols, the general trend is a lower toxicity in zebrafish than in rodents. Tween 80 is an exception to this trend because it is much more toxic to zebrafish. This could be because of its surfactant properties, a suggestion supported by the comparably high relative toxicity to zebrafish (98.33) that we find for another surfactant tested, sodium dodecyl sulphate (SDS). Our LC_50_ for SDS in 96 h exposure to zebrafish embryos was 3.6 mg/L. This is similar to the dose of SDS that causes pathological changes in the gills of the teleost *Thalassoma pavo* [47]. Copper also appears to interfere with ion transport in the gills [reviewed in: 48] as does lead [49]. The lower relative toxicity of colchicine to zebrafish has been previously reported [17]. The suggestion is that teleosts may have some protection by virtue of being unable to oxidise colchicine to the much more toxic oxycolchicine [17].

It is also possible that experimental methodology underlies some of the species differences found here. The standard error for the rodent LD_50_ values were not available in Toxnet or the Registry of Cytotoxicity. This is significant because error in the independent variable can have a significant effect on both slope and intercept. Other study-dependent influences on the data could include differences in exposure time, developmental stage, route of exposure between the zebrafish and rodent studies.

### Conclusions

Our findings show that the zebrafish embryo is a tool that offers potential in the evaluation of drug safety. However, we show that the predictivity varies between the class of compound studied. More work is required to examine how the covariance of zebrafish and rodent toxicity is influenced by such factors as compound type, absorption, metabolism and mechanism of toxicity.

## Supporting Information

Table S1 **Summary of compounds used in this study for toxicity evaluation in zebrafish embryo.**
(DOC)

Table S2 **Concentrations used in geometric series.**
(DOC)
