# Supplementary material for: Large-Scale Assessment of the Zebrafish Embryo as a Possible Predictive Model in Toxicity Testing
Source: PLoS One. 2011 Jun 28;6(6):e21076. doi: 10.1371/journal.pone.0021076 (PMC3125172; doi:10.1371/journal.pone.0021076)
Supplement: Table S1 — Summary of compounds used in this study for toxicity evaluation in zebrafish embryo. (DOC) [file pone.0021076.s001.doc]

# Supporting information

Table S1. **Summary of compounds used in this study for toxicity evaluation in zebrafish embryos.**

|  | **Name (synonym)** | **Supplier (City, Country)** | **Cat. number** | **Molecular weight ^(a)^ (g/L)** | **Water solubility (mg/L)** | **Chemical structure*** |
| --- | --- | --- | --- | --- | --- | --- |
| 1 | Aconitine | Sigma (Zwijndrecht, NL) | A8001 | 645.74 | 607**^(a)^** | Diterpene alkaloid |
| 2 | Atropine | BDH (Poole, UK) | 27276 | 289.38 | 2200**^(b)^** | Tropane alkaloid |
| 3 | Berberine chloride | Sigma (Zwijndrecht, NL) | B3251 | 371.81 | soluble**^(a)^** | Isoquinoline alkaloid |
| 4 | Colchicine | Sigma (Zwijndrecht, NL) | C3915 | 399.44 | 45000**^(b)^** | Alkaloid |
| 5 | Coniine | Sigma (Zwijndrecht, NL) | C9392 | 127.23 | 18000**^(b)^** | Piperidine alkaloid |
| 6 | α-Lobeline hydrochloride | Sigma (Zwijndrecht, NL) | 62630 | 373.92 | 25000**^(a)^** | Alkaloid |
| 7 | Morphine hydrochloride | Sigma (Zwijndrecht, NL) | 67357 | 423.89 | soluble**^(a)^** | Opiate alkaloid |
| 8 | Nicotine | Sigma (Zwijndrecht, NL) | N3876 | 162.26 | 1000000**^(b)^** | Solanaceous alkaloid |
| 9 | Quinine sulphate | Sigma (Zwijndrecht, NL) | Q0132 | 391.47 | 1430**^(b)^** | Cinchona alkaloid |
| 10 | (-)-Scopolamine hydrobromide trihydrate | Sigma (Zwijndrecht, NL) | S1875 | 438.31 | 666667**^(a)^** | Tropane alkaloid |
| 11 | Strychnine hydrochloride | Sigma (Zwijndrecht, NL) | S8753 | 370.87 | 28571**^(a)^** | Secologanin tryptamine alkaloid |
| 12 | Theobromine | Sigma (Zwijndrecht, NL) | T4500 | 180.16 | 500**^(a)^** | Xanthine alkaloid |
| 13 | (+)-Tubocurarine chloride hydrate | Sigma (Zwijndrecht, NL) | T2379 | 681.65 | 50000**^(a)^** | Isoquinoline alkaloid |
| 14 | Yohimbine hydrochloride | Sigma (Zwijndrecht, NL) | Y3125 | 390.90 | 7300**^(b)^** | Secologanin Tryptamine alkaloid |
| 15 | Amygdalin | Sigma (Zwijndrecht, NL) | A6005 | 457.43 | 83333**^(a)^** | Glycoside |
| 16 | Arbutin | Sigma (Zwijndrecht, NL) | A4256 | 272.25 | 50000**^(c)^** | Glycoside |
| 17 | Convallatoxin | Sigma (Zwijndrecht, NL) | C9140 | 550.64 | 500**^(b)^** | Cardiac glycoside |
| 18 | Coumarin | Sigma (Zwijndrecht, NL) | C4261 | 383644 | 1900**^(b)^** | Glycoside |
| 19 | Digitoxin | Sigma (Zwijndrecht, NL) | D5878 | 764.94 | 10^(a)^ | Cardiac glycoside |
| 20 | Gentamycin sulfate | Duchefa (Haarlem, NL) | G0124 | 575.67 | soluble**^(a)^** | Amino glycoside |
| 21 | Glycyrrhizin | Sigma (Zwijndrecht, NL) | 50531 | 839.96 | soluble**^(a)^** | Terpene glycoside |
| 22 | Hesperidin | Sigma (Zwijndrecht, NL) | H5254 | 610.56 | 57**^(d)^** | Flavanone glycoside |
| 23 | Kanamycin monosulfate | Duchefa (Haarlem, NL) | K0126.0005 | 582.6 | soluble**^(a)^** | Amino glycoside |
| 24 | Naringin | Sigma (Zwijndrecht, NL) | N1376 | 580.53 | 1000**^(b)^** | Flavanone glycoside |
| 25 | Neohesperidin | Sigma (Zwijndrecht, NL) | N1887 | 610.56 | 61**^(e)^** | Flavanone glycoside |
| 26 | Ouabain octahydrate | Sigma (Zwijndrecht, NL) | O3125 | 728.77 | 13333**^(a)^** | Cardiac glycoside |
| 27 | Phloridzin dihydrate | Sigma (Zwijndrecht, NL) | P3449 | 472.44 | 1300**^(a)^** | Glycoside |
| 28 | Rutin hydrate | Sigma (Zwijndrecht, NL) | R5143 | 610.52 | soluble**^(c)^** | Flavanone glycoside |
| 29 | Streptomycin sulfate | Sigma (Zwijndrecht, NL) | S6501 | 1457.38 | >20000**^(a)^** | Amino glycoside |
| 30 | Cadmium(II) chloride | Sigma (Zwijndrecht, NL) | 439800 | 479.67 | soluble**^(a)^** | Metal salt |
| 31 | Copper(II) nitrate trihydrate | Merck KGaA, Darmstadt, Germany | A911353 | 241.60 | 1378000**^(a)^** | Metal salt |
| 32 | Lead acetate trihydrate | BDH (Poole, UK) | 10142 | 379.33 | 625000**^(a)^** | Metal salt |
| 33 | Lithium chloride | JTB | 0157 | 42.39 | 769000**^(a)^** | Metal salt |
| 34 | Chloramphenicol | Sigma (Zwijndrecht, NL) | C0378 | 323.15 | 2500**^(a)^** | Alcohol |
| 35 | Ethanol | Merck KGaA, Darmstadt, Germany | 100971 | 46.07 | 1000000**^(b)^** | Alcohol |
| 36 | Glycerol | BDH (Poole, UK) | K33625960 | 92.11 | 1000000**^(b)^** | Sugar alcohol |
| 37 | Tween 80 | Sigma (Zwijndrecht, NL) | P1754 | 1310 | soluble**^(c)^** | Alcohol |
| 38 | Acetic acid | Merck KGaA, Darmstadt, Germany | K30123563 | 60.05 | 1000000**^(b)^** | Carboxylic acid |
| 39 | Salicylic acid | Sigma (Zwijndrecht, NL) | S0875 | 138.12 | 2240**^(b)^** | Carboxylic acid |
| 40 | Sodium oxalate | Sigma (Zwijndrecht, NL) | 71800 | 134 | 37000**^(a)^** | Carboxylic acid |
| 41 | Trichloroacetic acid | BDH, VWR Leuven, Belgium | 20741.290 | 163.38 | 10000000**^(a)^** | Carboxylic acid |
| 42 | Ampicillin sodium | Duchefa (Haarlem, NL) | A0104.0025 | 371.4 | 5000000**^(a)^** | Amide, penicillin G |
| 43 | Cyclophosphamide monohydrate, Cytoxan | Sigma (Zwijndrecht, NL) | C0768 | 279.1 | 40000**^(a)^** | Phosphor amide mustard |
| 44 | Paracetamol (Acetaminophen) | Sigma (Zwijndrecht, NL) | A7085 | 151.17 | 14000**^(b)^** | Amide |
| 45 | Phenacetin | Sigma (Zwijndrecht, NL) | 77440 | 179.22 | 766**^(b)^** | Amide |
| 46 | Benserazide hydrochloride | Sigma (Zwijndrecht, NL) | B7283 | 293.70 | 10000**^(c)^** | Hydrazine |
| 47 | Chlorpromazine hydrochloride | Sigma (Zwijndrecht, NL) | C8138 | 355.33 | 400000**^(c)^** | Phenothiazine |
| 48 | Isoniazid | Sigma (Zwijndrecht, NL) | I3377 | 137.14 | 140000**^(a)^** | Hydrazine |
| 49 | Phenelzine sulfate | Sigma (Zwijndrecht, NL) | P6777 | 234.27 | soluble**^(a)^** | Hydrazine |
| 50 | Ethambutol dihydrochloride | Sigma (Zwijndrecht, NL) | E4630 | 277.2 | 50000**^(a)^** | Ethylenediamines |
| 51 | Verapamil hydrochloride | Sigma (Zwijndrecht, NL) | 381175 | 491.1 | 83000**^(a)^** | Phenethylamine |
| 52 | Phenol | BDH (Poole, UK) | 10188 | 94.11 | 15000**^(a)^** | Carbolic acid |
| 53 | Sodium azide | Sigma (Zwijndrecht, NL) | S8032 | 65.01 | 417000**^(a)^** | Inorganic azide |
| 54 | Dimethyl sulfoxide | Sigma (Zwijndrecht, NL) | 60153 | 78.13 | 1000000**^(b)^** | Sulfoxide |
| 55 | Formaldehyde | Sigma (Zwijndrecht, NL) | 25254-9 | 30.03 | 400000**^(b)^** | Aldehyde |
| 56 | Phenformin hydrochloride | Sigma (Zwijndrecht, NL) | P7045 | 241.72 | soluble**^(a)^** | Biguanide |
| 57 | Ropinirole hydrochloride | Sigma (Zwijndrecht, NL) | R2530 | 296.84 | 400000**^(b)^** | Indole |
| 58 | Amitriptyline hydrochloride | Sigma (Zwijndrecht, NL) | A8404 | 313.87 | soluble**^(a)^** | Dibenzocycloheptene |
| 59 | Sodium dodecyl sulfate | LKB (Broma, Sweeden) | 1836 | 289.43 | 1000000**^(b)^** | Alkane sulfonate |
| 60 | Barbital sodium (Barbitalum natricum, Ph.Helv.) | BUFA (Ijsselstein, NL). | 175310 | 206.2 | 200000**^(a)^** | Barbiturate |

Key: (a), from [1]; (*), From the Pubchem database at http://pubchem.ncbi.nlm.nih.gov/. (b)**,** from Chemical Identification/Dictionary database at http://toxnet.nlm.nih.gov/cgi-bin/sis/search/; (c), from http://www.sigmaaldrich.com/catalog/DisplayMSDSContent.do/ ; (d), from [2]; (e), from [3].

Note: References

1. The Merck index (2006): An encyclopedia of chemicals, drugs, and biologicals. 14th ed: pp1-1156.

2. Sansone F, Rossi A, Del GP, De SF, Aquino RP, Lauro MR (2009) Hesperidin gastroresistant microparticles by spray-drying: preparation, characterization, and dissolution profiles. AAPS PharmSciTech 10: 391-401.

3. Kometani T, Nishimura T, Nakae T, Takii H, Okada S (1996) Synthesis of neohesperidin glycosides and naringin glycosides by cyclodextrin glucanotransferase from an alkalophilic Bacillus species. Biosci Biotechnol Biochem 60: 645-649.
