# Supplementary material for: Large-Scale Assessment of the Zebrafish Embryo as a Possible Predictive Model in Toxicity Testing
Source: PLoS One. 2011 Jun 28;6(6):e21076. doi: 10.1371/journal.pone.0021076 (PMC3125172; doi:10.1371/journal.pone.0021076)
Supplement: Table S2 — Concentrations used in geometric series. (DOC) [file pone.0021076.s002.doc]

# Supporting information

Table S2. **Concentrations used in geometric series.**

|  |  | **Concentrations in geometric series (mg/L)** | | | | | |
| --- | --- | --- | --- | --- | --- | --- | --- |
|  | **Compounds** | **C0** | **C1** | **C2** | **C3** | **C4** | **C5** |
| 1 | Aconitine | 0 | 50 | 100 | 200 | 400 | 800 |
| 2 | Atropine | 0 | 100 | 200 | 400 | 800 | 1600 |
| 3 | Berberine chloride | 0 | 50 | 100 | 200 | 400 | 800 |
| 4 | Colchicine | 0 | 10 | 20 | 40 | 80 | 160 |
| 5 | Coniine | 0 | 10 | 20 | 40 | 80 | 160 |
| 6 | α-Lobeline hydrochloride | 0 | 10 | 20 | 40 | 80 | 160 |
| 7 | Morphine hydrochloride | 0 | 1000 | 2000 | 4000 | 8000 | 16000 |
| 8 | Nicotine | 0 | 10 | 20 | 40 | 80 | 160 |
| 9 | Quinine sulfate | 0 | 30 | 60 | 120 | 240 | 480 |
| 10 | (-)-Scopolamine hydrobromide trihydrate | 0 | 1000 | 2000 | 4000 | 8000 | 16000 |
| 11 | Strychnine hydrochloride | 0 | 10 | 20 | 40 | 80 | 160 |
| 12 | Theobromine | 0 | 30 | 60 | 120 | 240 | 480 |
| 13 | (+)-Tubocurarine chloride hydrate | 0 | 100 | 200 | 400 | 800 | 1600 |
| 14 | Yohimbine hydrochloride | 0 | 10 | 20 | 40 | 80 | 160 |
| 15 | Amygdalin | 0 | 10 | 20 | 40 | 80 | 160 |
| 16 | Arbutin | 0 | 10 | 20 | 40 | 80 | 160 |
| 17 | Convallatoxin | 0 | 30 | 60 | 120 | 240 | 480 |
| 18 | Coumarin | 0 | 70 | 140 | 280 | 560 | 1120 |
| 19 | Digitoxin | 0 | 0.5 | 1 | 2 | 4 | 8 |
| 20 | Gentamycin sulfate | 0 | 100 | 200 | 400 | 800 | 1600 |
| 21 | Glycyrrhizin | 0 | 10 | 20 | 40 | 80 | 160 |
| 22 | Hesperidin | 0 | 10 | 20 | 40 | 80 | 160 |
| 23 | Kanamycin monosulfate | 0 | 250 | 500 | 1000 | 2000 | 4000 |
| 24 | Naringin | 0 | 50 | 100 | 200 | 400 | 800 |
| 25 | Neohesperidin | 0 | 10 | 20 | 40 | 80 | 160 |
| 26 | Ouabain octahydrate | 0 | 50 | 100 | 200 | 400 | 800 |
| 27 | Phloridzin dihydrate | 0 | 70 | 140 | 280 | 560 | 1120 |
| 28 | Rutin hydrate | 0 | 1000 | 2000 | 4000 | 8000 | 16000 |
| 29 | Streptomycin sulfate | 0 | 250 | 500 | 1000 | 2000 | 4000 |
| 30 | Cadmium(II) chloride | 0 | 10 | 20 | 40 | 80 | 160 |
| 31 | Copper(II) nitrate trihydrate | 0 | 6.25 | 12.5 | 25 | 50 | 100 |
| 32 | Lead acetate trihydrate | 0 | 10 | 20 | 40 | 80 | 160 |
| 33 | Lithium chloride | 0 | 1000 | 2000 | 4000 | 8000 | 16000 |
| 34 | Chloramphenicol | 0 | 100 | 200 | 400 | 800 | 1600 |
| 35 | Ethanol | 0 | 1000 | 2000 | 4000 | 8000 | 16000 |
| 36 | Glycerol | 0 | 2000 | 4000 | 8000 | 16000 | 32000 |
| 37 | Tween 80 | 0 | 100 | 200 | 400 | 800 | 1600 |
| 38 | Acetic acid | 0 | 50 | 100 | 200 | 400 | 800 |
| 39 | Salicylic acid | 0 | 7.5 | 15 | 30 | 60 | 120 |
| 40 | Sodium oxalate | 0 | 100 | 200 | 400 | 800 | 1600 |
| 41 | Trichloroacetic acid | 0 | 20 | 40 | 80 | 160 | 320 |
| 42 | Ampicillin sodium | 0 | 250 | 500 | 1000 | 2000 | 4000 |
| 43 | Cyclophosphamide monohydrate | 0 | 1000 | 2000 | 4000 | 8000 | 16000 |
| 44 | Paracetamol | 0 | 100 | 200 | 400 | 800 | 1600 |
| 45 | Phenacetin | 0 | 50 | 100 | 200 | 400 | 800 |
| 46 | Benserazide hydrochloride | 0 | 250 | 500 | 1000 | 2000 | 8000 |
| 47 | Chlorpromazine hydrochloride | 0 | 1 | 2 | 4 | 8 | 16 |
| 48 | Isoniazid | 0 | 200 | 400 | 800 | 1600 | 3200 |
| 49 | Phenelzine sulfate | 0 | 5 | 10 | 20 | 40 | 80 |
| 50 | Ethambutol dihydrochloride | 0 | 1000 | 2000 | 4000 | 8000 | 16000 |
| 51 | Verapamil hydrochloride | 0 | 10 | 20 | 40 | 80 | 160 |
| 52 | Phenol | 0 | 10 | 20 | 40 | 80 | 160 |
| 53 | Sodium azide | 0 | 0.5 | 1 | 2 | 4 | 8 |
| 54 | Dimethyl sulfoxide | 0 | 2000 | 4000 | 8000 | 16000 | 32000 |
| 55 | Formaldehyde | 0 | 2 | 4 | 8 | 16 | 32 |
| 56 | Phenformin hydrochloride | 0 | 100 | 200 | 400 | 800 | 1600 |
| 57 | Ropinirole hydrochloride | 0 | 100 | 200 | 400 | 800 | 1600 |
| 58 | Amitriptyline hydrochloride | 0 | 2 | 4 | 8 | 16 | 32 |
| 59 | Sodium dodecyl sulfate | 0 | 1 | 2 | 4 | 8 | 16 |
| 60 | Barbital sodium | 0 | 500 | 1000 | 2000 | 4000 | 8000 |

For each compound, a geometric series of concentrations (C0 – C5) was used, based on the results of the logarithmic range-finding series.
